# Difficult Intubation Alert Is Associated With a Reduced Incidence of Difficult Intubation

**DOI:** 10.7759/cureus.72625

**Published:** 2024-10-29

**Authors:** Anna M Budde, Andrea L Barrett, Ashley C Benner, Lauren B Gunn-Sandell, Alexander M Kaizer

**Affiliations:** 1 Anesthesiology, University of Minnesota School of Medicine, Minneapolis, USA; 2 Occupational Therapy, University of Minnesota School of Medicine, Minneapolis, USA; 3 Clinical and Translational Science Institute, University of Minnesota School of Medicine, Minneapolis, USA; 4 Biostatistics and Informatics, Colorado School of Public Health, University of Colorado-Anschutz Medical Campus, Aurora, USA

**Keywords:** academic anesthesiology, anesthesia documentation, difficult airway intubation, difficult airway management, quality improvement project

## Abstract

Introduction: Difficult or failed intubation significantly increases the risk of morbidity and mortality. Documentation of a prior difficult or failed tracheal intubation is a strong predictor of future difficult intubation.

Methods: We undertook a quality improvement project to create a redesigned difficult intubation alert with increased visibility in our electronic health record. We sought to determine whether this redesigned alert would be associated with a reduced incidence of difficult intubations. After reviewing many intubation procedure notes, we chose the following criteria to define a predicted future difficult intubation: requiring an awake technique, ease of intubation procedure charted as “difficult” or “unable”, procedure requiring flexible bronchoscopy, a procedure requiring three or more attempts, and intubation with a grade three or four view during laryngoscopy. Patients meeting one or more of the above criteria were included in our study. An intervention was implemented which consisted of the introduction of a new difficult intubation alert that could easily be applied to a patient’s chart by anyone on the anesthesia team. Further, if the anesthesia clinician filling out the intubation procedure note charted an intubation procedure as “difficult” or “unable”, they were prompted by a pop-up asking if difficult intubation should be added to the patient’s problem list. If yes was clicked, the electronic alert was activated, and a large red banner appeared. Outcomes included the number of patients who had the difficult intubation label in the pre-intervention period, the number of patients who had the new difficult intubation alert in the post-intervention period, the number of records with ease of intubation procedure charted as “difficult” or “unable”, the number of records requiring three or more attempts at intubation, and the number of records with grade three or four view charted at intubation.

Results: There was an expected increase in the application of the difficult intubation alert from 9% of patients with a difficult intubation label in the pre-intervention period to 38% with the redesigned alert in the post-intervention period which was statistically significant (p<0.001). In the 21 months prior to the introduction of the alert, our screening process identified 988 records as predicted difficult intubations. Of these, 672 (68%) were charted by the intubating clinician as actual difficult intubations with 32% not being recorded as difficult. During the 20 months after the end of the interim period, the screening process identified 976 predicted difficult intubations with intubating anesthesia clinicians charting 416 (42%) of them as actual difficult intubations and 58% found not to be difficult. This reduction in monthly median percent of actual difficult intubations was statistically significant (p<0.001).

Conclusions: The introduction of a difficult intubation alert at our institution was associated with a reduced incidence of difficult intubation.

## Introduction

A difficult intubation is defined by the American Society of Anesthesiologists as a clinical situation in which a physician trained in anesthesia care has difficulty with tracheal intubation, requires multiple attempts at tracheal intubation, or tracheal intubation fails [1]. Difficult or failed tracheal intubation poses a high risk of hypoxia, cardiac arrest, and death [2]. Anesthesiologists have limited time to successfully manage an airway. With each attempted instrumentation of the airway, there is an increased risk of airway trauma which reduces the likelihood of success with each attempt.

While many bedside screening tools are utilized to predict difficult intubations, their sensitivity and specificity are limited and many patients with difficult intubation are missed [3]. However, documentation of a previous difficult or failed tracheal intubation is a strong predictor of subsequent difficult tracheal intubation [4]. Structured notes in the electronic health record have been shown to facilitate clinician documentation of difficult intubations [5]. Therefore, documentation of prior intubation difficulty is critically important information to transmit to future care teams to ensure safe patient care [6]. Additionally, a recent study demonstrated complications in patients with predicted difficult intubations were 45 times more common compared with patients with predicted easy intubations, highlighting the importance of difficult intubation prediction [7].

A difficult intubation label existed within our electronic health record (Epic Systems Corporation, Verona, WI, USA); however, this label was poorly visible and often missed by anesthesia team members. Therefore, a quality improvement initiative was undertaken with the goal of improving communication by creating an improved “prior difficult intubation” alert in our electronic health record. We hypothesized that by creating a clearly visible alert forewarning anesthesiologists about a patient’s history of difficult intubation rather than relying on an anesthesiologist reviewing prior airway records, we would note an associated reduction in the incidence of difficult intubation. We hypothesized this reduction would be due to better preparation and awareness of which method and equipment were successful in past intubations.

## Materials and methods

After reviewing many intubation procedure notes, we chose the following criteria to define a predicted future difficult intubation: requiring an awake technique, ease of intubation procedure charted as “difficult” or “unable”, procedure requiring flexible bronchoscopy, a procedure requiring three or more attempts, and intubation with a grade three or four view during laryngoscopy. We electronically screened all intubation procedure notes for operating room intubations in adult and pediatric patients performed at our institution and all affiliated institutions throughout the state of Minnesota between January 1, 2020, and June 30, 2023, for those meeting one or more of the above criteria. If one or more of the difficult intubation criteria were met, that patient was included in our analysis. This study was submitted to our local institutional review board and determined to be non-human research.

**Figure 1:**
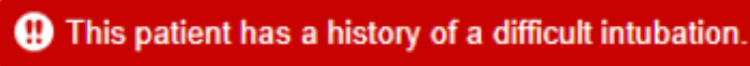
Difficult Intubation Alert

An intervention was implemented in October 2021 which consisted of the introduction of a new difficult intubation alert that could easily be applied to a patient’s chart by anyone on the anesthesia team. Further, if the anesthesia clinician filling out the intubation procedure note charted an intubation procedure as “difficult” or “unable”, they were prompted by a pop-up asking if difficult intubation should be added to the patient’s problem list. If yes was clicked, the electronic alert was activated, and a large red banner appeared. This banner would then appear on any subsequent anesthesia record at the time it was initially opened by anyone on the anesthesia team. The banner is shown in Figure 1.

Once applied, the difficult intubation alert was present and did not need to be reapplied. During the month of October, 2021, we rolled out the new electronic alert and thus excluded all intubations performed during this month as the interim period. The criteria used to define a predicted future difficult intubation which were used to include patients in our analysis were the same throughout the study in both pre and post-intervention time periods.

Outcomes included the number of patients who had the difficult intubation label in the pre-intervention period, the number of patients who had the new difficult intubation alert in the post-intervention period, the number of records with ease of intubation procedure charted as “difficult” or “unable”, the number of records requiring three or more attempts at intubation, and the number of records with grade three or four view charted at intubation. An actual difficult intubation was defined as one charted as "difficult" or "unable" by the anesthesia team performing the intubation. This criteria was the same in pre and post-implementation periods. Data were aggregated by month and then summarized and stratified by pre or post October 2021 implementation. Proportions of each outcome were then calculated out of the total number of predicted difficult intubations by month as patient counts varied each month. This generated a proportional value as the percent of predicted difficult intubations for each of the unique outcomes. The bivariate test comparing the pre-percentage and post-percentage outcomes was the Wilcoxon rank sum test. 

In order to account for varying counts in the monthly underlying difficult intubation populations, a Poisson model with an offset for monthly population size was fitted for each of the three outcomes. Results are displayed as incidence rate ratios of the post-implementation era in reference to the pre-implementation era.

## Results

Among those patients identified by our screening process as predicted future difficult intubations, 9% had a difficult intubation label in their electronic medical record in the pre-intervention period. In the post-intervention period, 38% of patients identified as predicted future difficult intubations had the redesigned difficult intubation alert applied to their electronic health record. This increase in the monthly proportion of patients with a difficult intubation alert was statistically significant (p<0.001).

**Figure 2:**
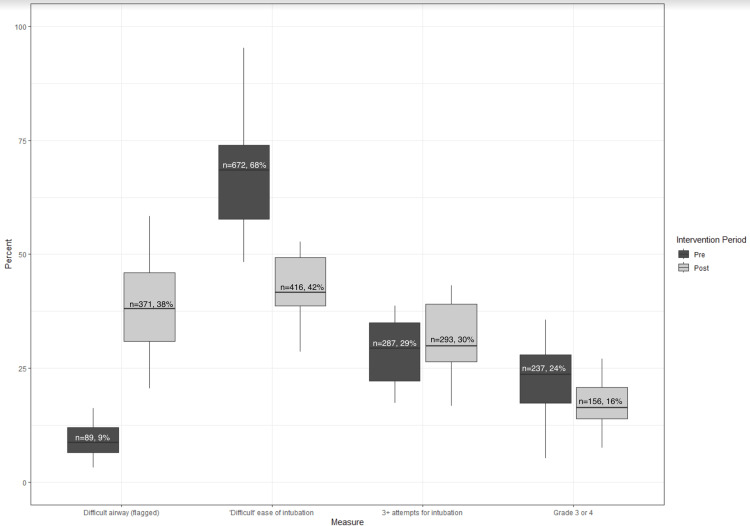
Difficult Intubation Metrics in Pre- and Post-Intervention Periods

In the 21 months prior to the introduction of the alert, our screening process identified 988 records as predicted difficult intubations. Of these identified by our screening process, 672 (68%) were also charted by the intubating clinician as actual difficult intubations with 32% not recorded as difficult. The alert was introduced on October 1, 2021. This was followed by a one-month educational period. During the 20 months after the end of this period, the screening process identified 976 predicted difficult intubations with intubating anesthesia clinicians charting 416 (42%) of them as actual difficult intubations and 58% not recorded as difficult. This reduction in the monthly median percent of difficult intubations that were charted as difficult by the anesthesia clinicians was statistically significant (p<0.001). These data are displayed graphically in Figure 2.

Additionally, the monthly median percent of grade view of cords charted as three or four also decreased, from 24% to 16% after the alert intervention and this was statistically significant (p=0.03).

However, there was no evidence of a difference in the monthly median percent of three or more intubation attempts between the pre- and post-eras, from 29% to 30% (p=0.30).

These results are displayed in Table 1.

**Table 1:** Difficult Intubations in Pre- and Post-Intervention Periods *Median (interquartile range)

Characteristic	Overall, N=41*	Pre-Intervention, N=21*	Post-Intervention, N=20*	p-value
% Difficult Intubation Alert	21 (9, 38)	9 (6, 12)	38 (31, 46)	<0.001
% "Difficult" Ease of Intubation	52 (42, 68)	68 (58, 74)	42 (39, 49)	<0.001
% 3+ Attempts for Intubation	30 (26, 37)	29 (22, 35)	30 (26, 39)	0.3
% Grade View of Cords, 3 or 4	20 (16, 25)	24 (17, 28)	16 (14, 21)	0.03

A Poisson regression was additionally performed. The rate of intubations in the post-implementation period classified by the intubating clinician as difficult was 0.64 times that of the pre-implementation period (i.e., 36% lower) after controlling for the underlying monthly population variation, which was statistically significant.

The rate of three or more intubation attempts in the post-implementation era was 1.06 times that of the pre-implementation era, controlling for the underlying monthly population variation, and this was not statistically significant. The rate of grade three or four view in the post-implementation era was 0.74 times that of the pre-implementation era (i.e., 26% lower), controlling for the underlying monthly population variation, and this was statistically significant. These results are shown in Table 2.

**Table 2:** Poisson Regression Results *IRR: incidence rate ratio

Characteristic	IRR*	95% CI	p-value
‘Difficult’ Ease of intubation	0.64	(0.57, 0.73)	<0.0001
3+ Attempts of Intubation	1.06	(0.9, 1.24)	0.5
Grade 3 or 4 View	0.74	(0.61, 0.9)	0.003

. 

## Discussion

We describe a quality improvement project aiming to improve communication around prior difficult intubations using a redesigned difficult intubation alert in our electronic health record. Consensus recommendations of the Canadian Airway Focus Group list prior intubation difficulty as more predictive of future difficulty than physical exam, which highlights the importance of clearly communicating information about prior intubation difficulty [8]. Among those patients included in our analysis with predicted future difficult intubations, we noted an expected increase in the application of the difficult intubation alert in our post-intervention period as compared with our pre-intervention period. We hypothesize this increase in the utilization of the difficult intubation alert was due to reduced barriers to its application in addition to a prompt asking the intubating clinician whether the difficult intubation alert should be added if the clinician charted an intubation procedure as “difficult” or “unable”. Difficult intubation alerts have been implemented successfully in other institutions to optimize communication about a prior episode of intubation difficulty [9]. Additional studies describe broader difficult intubation quality improvement projects which include an alert in the electronic medical record to ensure communication of prior intubation difficulty [10, 11].

While it is clear that alerting future care teams to a history of intubation difficulty is important, there is a paucity of literature describing improved patient outcomes related to a difficult intubation alert. In one study, investigators describing a quality improvement project aiming to improve the management of patients with difficult intubation found a decrease in the need for emergency surgical airway. An important component of the quality improvement initiative included an alert in the electronic health record [12]. Our findings include a reduction in actual difficult intubations between pre- and post-intervention time periods which we defined in both periods as intubation charted by the anesthesia team as “difficult” or “unable”. These findings demonstrate that the introduction of an improved difficult intubation alert at our institution was associated with a reduction in actual recorded difficult intubations. We additionally found a reduction in grade three and four laryngoscopic view in the post-intervention period as compared with the pre-intervention period. We found no difference in the rates of three or more attempts at airway management.

We hypothesize that being alerted to a patient’s history of difficult intubation allowed anesthesiologists to better prepare for airway management. Additionally, the availability of the prior record detailing the equipment utilized when the intubation was found to be difficult likely allowed the anesthesiologist to optimize their plan and avoid pitfalls that may have occurred in the past. Additional backup supplies or skilled personnel are also likely to have been gathered for management of an anticipated difficult intubation which would not have practically been available in the case of an unanticipated difficult intubation [13].

We acknowledge the limitations of this study including the fact that while we establish an association between increased use of a difficult intubation alert and decreased incidence of clinicians charting intubation as difficult, we are unable to identify which specific factors may have led to this association. Therefore, to further understand why this association was found we identified a sample of patients who were intubated in the pre-intervention period and again in the post-intervention period. We compared intubation techniques and reported intubation difficulty, and we determined whether a difficult intubation alert had been applied to the patient’s chart. We found several examples of patients who lacked a difficult intubation label in the pre-intervention period and subsequently had a redesigned difficult intubation alert in the post-intervention period. Comparing intubation notes in these patients revealed that where the intubating clinician had charted the intubation as “difficult” in the pre-intervention period the intubation procedure was no longer charted as “difficult” in the post-intervention period. The most common findings in these charts were a technique change such as a change in video laryngoscope blade, a change to fiberoptic bronchoscopy technique, or a change to an awake intubation approach. We hypothesize that being alerted to the history of intubation difficulty via the difficult intubation alert allowed anesthesiologists to better prepare for these intubations and therefore the incidence of intubation difficulty was reduced. We hypothesize that a change in technique such as utilization of a different video laryngoscope blade could explain our findings of reduced grade three or four laryngoscopic view. While we cannot identify specifically why there was no difference found in the rates of intubations requiring three or more attempts between time periods, we suggest that as a teaching institution, some attempts may be made by learners which could explain these findings.

We present this simple intervention and suggest it to other institutions to reduce the incidence of intubation difficulty and improve patient care. Other institutions have introduced similar alerts to facilitate documentation of difficult intubations, including some studies of the utilization of an automated difficult airway alert based on pre-specified criteria [14]. However, no other studies have been published describing a reduction in intubation difficulty associated with improved labeling of difficult intubations.

## Conclusions

In this quality improvement initiative, we noted an increase in the application of a difficult airway alert to patients we screened as having a predicted future difficult intubation was associated with a reduction in actual difficult intubations as charted by clinicians. We plan to continue to educate members of our anesthesia team about the importance of labeling patients with difficult intubation in hopes that we see a further reduction in the incidence of difficult intubation at our institution. Further studies should attempt to clarify specifically which factors brought about this association. Additionally, studies to assess whether there is a decrease in morbidity and mortality associated with improved communication around difficult intubations would be useful.
